# Residency Match Trends, Racial Disparity, and Matching Amid a Pandemic

**DOI:** 10.1016/j.adro.2020.11.005

**Published:** 2020-11-23

**Authors:** April Vassantachart, Lindsay Hwang, Andrew Vassantachart, Richard Jennelle

**Affiliations:** a Department of Radiation Oncology, LAC + USC Medical Center, Los Angeles, California; b Loma Linda University Medical Center, Loma Linda, California

## Abstract

**Purpose:**

Radiation oncology has been facing an evolving crisis in recruitment for several years, and the events of 2020 to 2021 will certainly add to that crisis with the urgency of addressing systemic racial injustice amid a global pandemic. The purpose of this study is to examine applicant data to gain insight on residency match trends and evaluate these findings within the backdrop of a novel match year.

**Methods and Materials:**

National Residency Matching Program (NRMP) data between 2009 and 2020 were assessed for the number of applicants, programs, and positions available, number of ranked applicants needed to fill positions, and successfully matched applicant data. Additionally, Electronic Residency Application Service data were evaluated for race/ethnicity identification among applicants.

**Results:**

The number of applicants who ranked radiation oncology as their preferred specialty has declined for 3 consecutive years from 223 in 2017 to 155 in 2020. In 2020 the applicant-to-position ratio was at an all-time low at 0.82, and the unmatched position rate increased to 18.5%. The percentage of Black or African American applicants applying to radiation oncology has also declined to 4.9%, and this population represents 7.2% of all applicants. The number of ranked applicants needed to fill the available radiation oncology PGY2 positions increased from 4.0 in 2010 to 6.0 in 2020.

**Conclusion:**

Declining interest in radiation oncology among applicants, and an even further decline of black applicants, along with the challenges of interview and travel restrictions during the pandemic provide heightened concern for this year’s match. Innovative efforts to expand the reach of radiation oncology to prospective applicants is needed to engage diverse, bright, and committed students for the continued progress of radiation oncology and most importantly, our patients.

Radiation oncology has been facing an evolving crisis in recruitment for several years, and the events of 2020 to 2021 will certainly add to that crisis with the urgency of addressing systemic racial injustice amid a global pandemic. National Residency Matching Program (NRMP) data continue to show a decline in applicants who rank radiation oncology as their preferred specialty for 3 consecutive years (Fig 1).^1^ In 2019, there was a startling decline with fewer applicants than residency positions offered. The steep decline continued in 2020 with an applicant-to-position ratio of 0.82. In 2020, the unmatched position rate increased to 18.5%, up from an all-time high of 14.5% in 2019.^1^ A summary of the NRMP data during the past decade is outlined in Table 1. Although interest has been declining, NRMP data on accepted radiation oncology residents continue to show high-quality residents with mean United States Medical Licensing Examination step scores, number of research presentations/publications, and Alpha Omega Alpha Honor Medical Society students greater than the average applicant data and have been on the uptrend until 2020 (Table 2).^1^^,^^2^

**Figure 1:**
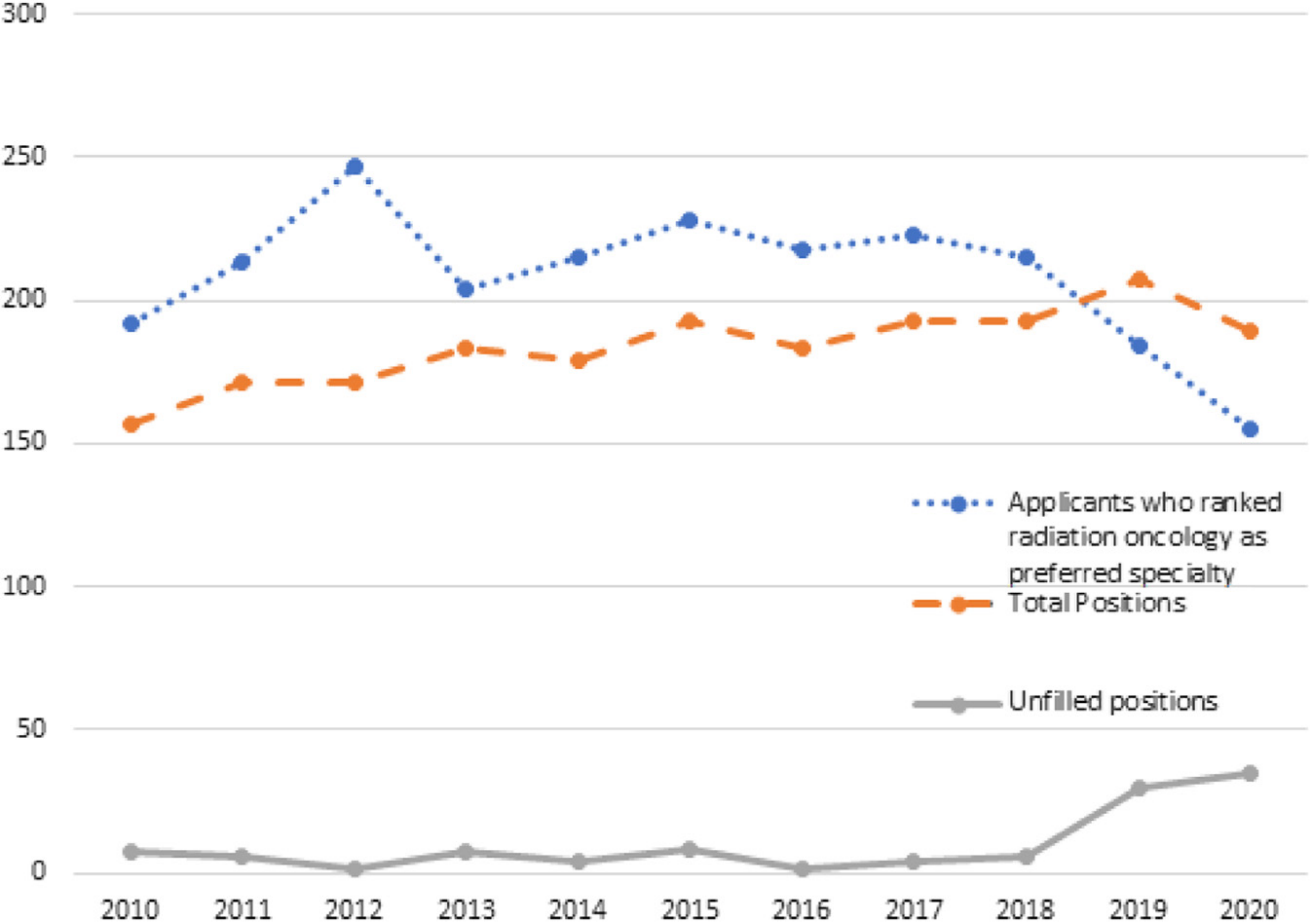
Applicant and position trends from 2010 to 2020.

**Table 1:** NRMP charting outcomes 2010 to 2020

	2010	2011	2012	2013	2014	2015	2016	2017	2018	2019	2020
No. of											
programs	78	82	85	93	87	90	85	91	93	92	88
Positions	157	171	171	183	179	193	183	193	193	207	189
Unfilled positions	7	6	1	7	4	8	1	4	6	30	35
Applicants who ranked RO as preferred specialty (% of all applicants)	192 (0.6%)	213 (0.7%)	247 (0.8%)	204 (0.6%)	215 (0.6%)	228 (0.7%)	218 (0.6%)	223 (0.6%)	215 (0.6%)	184 (0.5%)	155 (0.4%)
US seniors (% of US seniors)	163 (1.0%)	181 (1.1%)	217 (1.3%)	166 (0.9%)	188 (1.1%)	192 (1.1%)	186 (1.0%)	196 (1.1%)	190 (1.0%)	160 (0.8%)	122 (0.6%)
Independent∗ (% of independent)	29 (0.2%)	32 (0.2%)	30 (0.2%)	38 (0.2%)	27 (0.2%)	36 (0.2%)	32 (0.2%)	27 (0.2%)	25 (0.1%)	24 (0.1%)	33 (0.2%)
Applicant-to-position ratio for applicants who ranked RO as preferred specialty	1.22	1.25	1.44	1.11	1.20	1.18	1.19	1.16	1.11	0.89	0.82
Matched applicants	150	165	170	176	175	185	182	189	187	177	154
US senior	137	155	168	151	169	179	169	180	177	160	122
Other US graduate	5	9	2	19	1	5	5	3	4	5	8
DO graduate	5	1	0	2	4	1	4	1	3	5	7
US IMG	1	0	0	3	1	0	1	0	0	0	1
Non-US IMG	2	0	0	1	0	0	3	5	3	7	16
Unmatched US seniors who ranked RO as their only specialty	20 (14.5%)	23 (14.1%)	13 (15.3%)	5 (6.3%)	5 (6.0%)	3 (3.9%)	6 (7.9%)	3 (3%)	4 (3.9%)	1 (1.0%)	0 (0%)
Average number of ranked applicants needed to fill position											
PGY1	5.6	6.7	6.1	3.6	7.7	7.8	7.3	4.5	6.1	3.4	6.1
PGY2	4.0	3.9	3.3	5.8	4.9	5.2	5.4	5.7	5.9	6.7	6.0

*Abbreviations:* DO = osteopathic medicine; IMG = international medical graduate; NRMP = National Residency Matching Program; PGY = postgraduate year; RO = radiation oncology.

∗ Independent applicants include US graduates (nonseniors), DO graduates, and IMGs.

**Table 2:** Matched applicant data

	2009	2011	2014	2016	2018	2020
Contiguous ranked programs, mean						
RO	10.3	10.8	11.2	11.6	12.3	14.0
ALL	9.4	10.4	11.5	11.8	12.3	12.5
USMLE step 1 score, mean						
RO	238	240	241	247	247	243
ALL	225	226	230	233	233	234
USMLE step 2 score, mean						
RO	241	244	248	251	253	250
ALL	231	235	243	245	246	247
Abstracts, presentations, and publications, mean No.						
RO	8.0	8.3	12.2	12.7	15.6	18.3
ALL	2.8	3.2	4.2	4.7	5.7	6.9
AOA students, %						
RO	35.1	31.2	23.6	27.5	35.2	22.3
ALL	15.3	15.0	16.0	17.3	17.0	16.7
Students from top 40 NIH-funded medical schools, %						
RO	54.5	45.5	48.8	41.6	40.0	46.4
ALL	35.0	34.4	32.7	32.1	31.9	31.0
MD/PhD students, %						
RO	21.6	22.1	23.0	24.8	20.8	19.2
ALL	4.2	4.4	3.9	4.1	4.0	3.7

*Abbreviations:* ALL = average of all applicants; AOA = Alpha Omega Alpha Honor Medical Society; NIH = National Institutes of Health; RO = radiation oncology; USMLE = United States Medical Licensing Examination.

The reasons for decreased interest in the field among applicants is still under investigation. However, within the radiation oncologist community concern regarding an oversupply of practitioners may be responsible. Fear of physician oversupply increased from 34% in 2012 to 53% in 2017 according to the most recent workforce survey, and 52% of senior residents report the current job market as more competitive compared with former residents.^3^^,^^4^ In the workforce survey, the percentage of respondents who reported vacancies in their practice decreased by 4.1% from 19.3% in 2012 to 15.2% in 2017.^3^ This is in contrast with the number of residency program positions, which increased by 31.8% from 157 in 2010 to 207 in 2019 and more than doubled from a nadir of 93 in 2001.^1^ This perceived oversupply also has a geographic component. The greatest growth in residency training positions is in large programs with >12 trainees located in top metropolitan areas.^5^ Anonymous survey results from graduated radiation oncology residents show that nearly two-thirds of graduates prefer jobs in large cities with regional jobs in the West and South sought after the most and least, respectively.^6^ Although the largest proportion of job vacancies was reported at urban practices (17.1%) compared with suburban (6.6%) and rural practices (8.3%), less than half of available jobs are located in large cities and a majority of job openings overall are located in the Midwest and South.^3^^,^^4^^,^^6^^,^^7^ So at least part of the fear of oversupply may reflect perception rather than reality as job listings on the American Society for Radiation Oncology (ASTRO) Career Center website continue to show more listings than graduating residents.^4^^,^^7^ Importantly, 75% of recent graduates received a job offer in their preferred geographic region.^6^ In addition, shifts away from private practices toward academic and satellite centers in addition to changing compensation plans contribute to evolving job quality metrics within the field.^3^^,^^4^ The mismatch between available urban jobs and the greater proportion of graduates who prefer to work in large cities as well as the changing landscape of job metrics may contribute to insecurity for prospective applicants.

Even more devastating than the overall declining interest in radiation oncology among prospective applicants is the precipitous drop in black or African American applicants. Non-Hispanic blacks (NHBs) comprise 13% of the total US population but represent only 7.2% of the total residency applicant pool in 2019.^8^, ^9^, ^10^ Radiation oncology is doing a particularly poor job of improving racial diversity with only 4.7% of applicants applying to radiation oncology identifying as NHB (Fig 2).^8^ Racial concordance between physician and patient has been shown to improve information exchange and patient participation.^11^ By failing to recruit a diverse physician population, our field contributes to the systemic racism that promotes health care disparities.^12^^,^^13^ NHBs have the highest rate of cancer mortality despite having a lower incidence of cancer compared with non-Hispanic whites, and their disproportionate marginalization in clinical trials is a continuing area of concern.^9^^,^^10^ Structural racism has excluded NHB applicants within the medical field and urgent action is needed to increase black physicians in radiation oncology, as exclusion of any exceptional candidate is a loss of intellect, ideas, and innovation within our field. NHB representation is paramount within the workforce to pioneer the dissolution of systemic racism and to increase the intellectual breadth brought only by a diverse workforce.^12^^,^^13^

**Figure 2:**
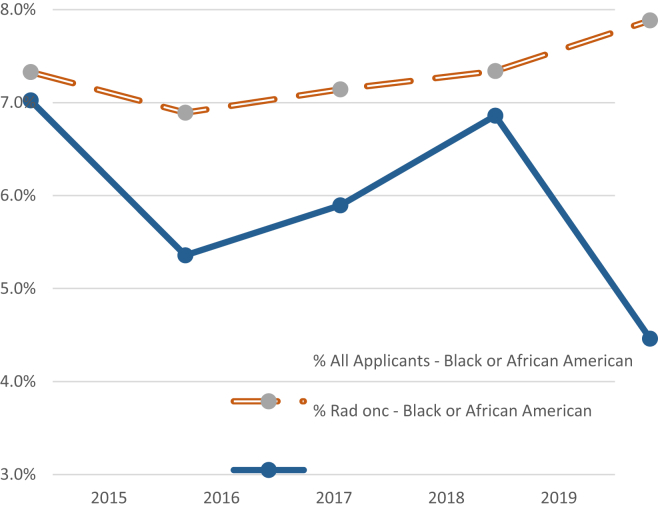
Percent of black or African American applicants between all applicants and radiation oncology applicants.

There is already an underlying concern for filling the available radiation oncology residency positions. Current trends show both US and international graduates are applying and interviewing at more programs, and the number of contiguously ranked radiation oncology programs has increased since 2009 from 10.3 to 14.0 in 2020.^1^^,^^14^ This, however, is misleading, as the average number of US medical graduate applicants per radiation oncology program has decreased from 167 in 2017 to 113.3 in 2020.8 Although exposure to more programs may be beneficial for applicants, it challenges residency programs to properly identify the appropriate candidates and number of candidates to interview. Ultimately, this could result in increased invitations from residency programs to the same pool of standard “highly qualified” applicants, which could lead to both an increase in unmatched positions and unmatched applicants, and is likely to further antagonize the acceptance of black applicants.

Owing to the coronavirus disease 2019 (COVID-19) pandemic and the associated restrictions on travel and away rotations, few applicants will be able to visit programs. These “audition” rotations help guide both the applicant and program rank processes through building relationships. We know from personal experience that candidates who successfully rotate with us are more likely to be considered favorably despite weaknesses in their traditional application and that this can be an effective tool to counter systemic bias. Data collected from the student doctor network online forum and its radiation oncology match spreadsheet have shown that applicants complete an average of 3 rotations in radiation oncology (including their home program) and 51.5% match to 1 of those programs.^15^ It is possible that changes to the interview and away rotation process this coming year will further increase the number of programs that applicants both apply to and accept interviews from due to decreased exposure to programs as well as decreased time and travel commitments needed to interview. With less opportunity to judge the whole candidate through personal interactions, we fear this year may have unintended consequences that further reinforce structural racism. From an institution perspective, the number of ranked applicants needed to fill the available radiation oncology PGY2 positions has been increasing from 4.0 in 2010 to 6.0 in 2020 (Fig 3).^1^ With a trend toward decreased applicant interest added to the pandemic-associated changes, estimating the number of applicant interviews needed to fill radiation oncology positions will be even more difficult.

**Figure 3:**
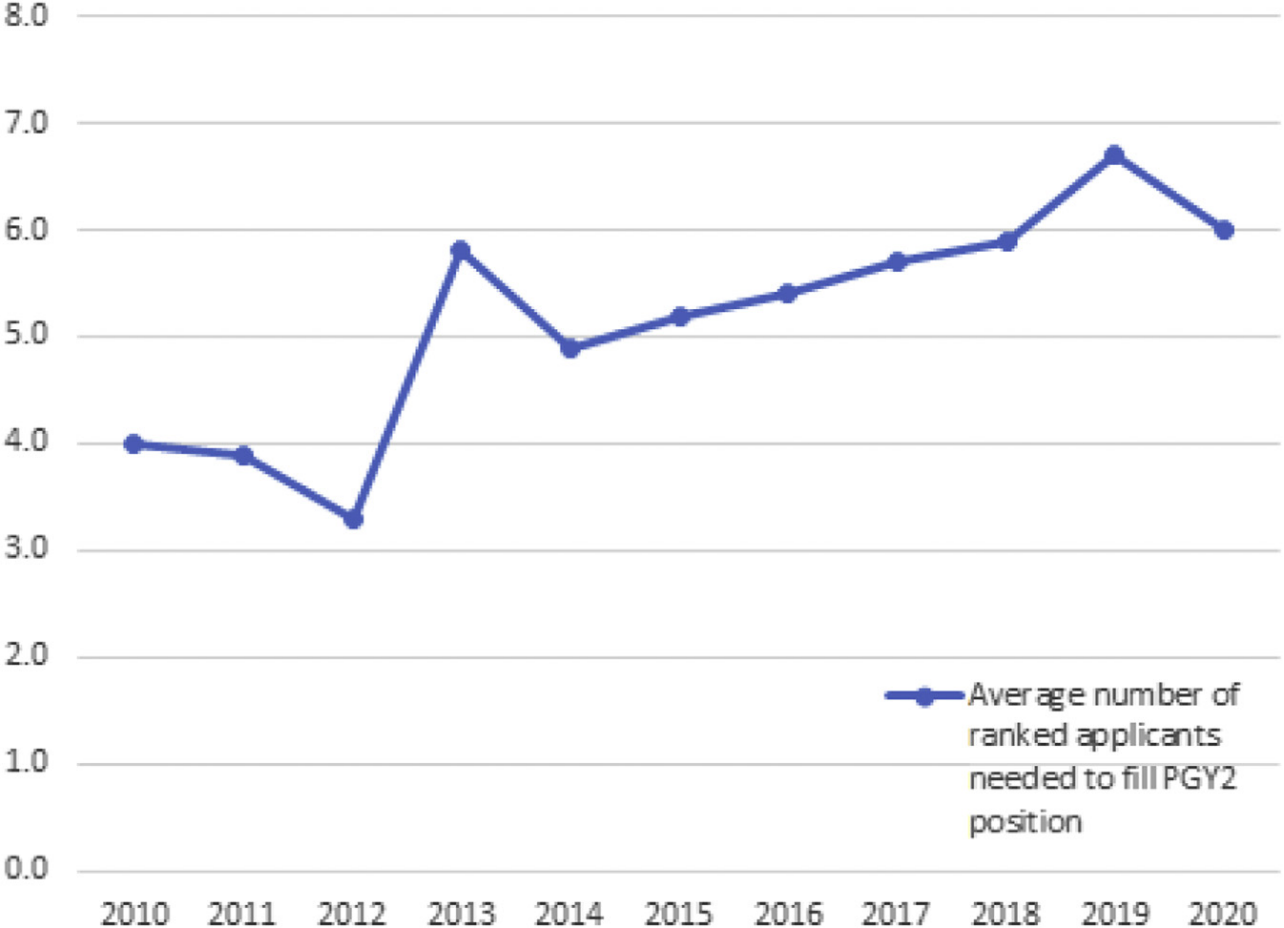
Number of ranked applicants needed to match.

Efforts to engage potential applicants interested in radiation oncology span several platforms including social media and online resources. At the 2019 annual ASTRO meeting, “supertweeters” in radiation oncology were recognized reflecting the growing importance of a strong online presence. In addition, multiple institutions have collaborated to create a virtual radiation oncology elective, the Radiation Oncology Virtual Education Rotation. This allows interested medical students to review prerecorded videos and interact with radiation oncology faculty from other institutions, which may be paramount particularly for students from institutions that do not have an academic radiation oncology department or those who have a geographic preference. In addition, virtual meet and greet sessions have been posted such that applicants can interact with current residents and faculty at participating institutions and can allow for 1:1 mentorship. Continued efforts to expand the reach of radiation oncology in medical schools are needed, perhaps now more than ever. To counteract systemic racism, we need to be involved with organizations including the Student National Medical Association and actively engage in recruitment at historic black schools. As a field, we must demonstrate active interest and effort as we strive toward a diverse physician workforce.

Innovative leadership by individual programs and the radiation oncology field as a whole is needed to recruit future applicants and especially our future black colleagues. Although applicant data continue to show high-quality candidates, it is important to continue evaluating the needs of the current workforce and adjust the number of residency positions accordingly. Addressing the interests of the medical student population (especially the black medical student population) to engage diverse, bright, and committed students for the continued progress of radiation oncology is paramount for our field, but absolutely essential for our patients.

## References

[1] National Resident Matching Program Report archives: 2009-2020 Main residency match. http://www.nrmp.org/report-archives.

[2] Chowdhary M, Parikh SD, Lee A, Tendulkar RD, Royce TJ. Radiation oncology resident quality by national resident matching program metrics from 2007 to 2018 [e-pub ahead of print]. *Int J Radiat Oncol Biol Phys.* https://doi.org/10.1016/j.ijrobp.2020.08.062, accessed December 7, 2020. 10.1016/j.ijrobp.2020.08.062 32891796

[3] Fung C.Y., Chen E., Vapiwala N. (2019). The American Society for Radiation Oncology 2017 radiation oncologist workforce study. Int J Radiat Oncol Biol Phys.

[4] Kahn J., Goodman C.R., Albert A. (2020). Top concerns of radiation oncology trainees in 2019: Job market, board examinations, and residency expansion. Int J Radiat Oncol Biol Phys.

[5] Chowdhary M., Sen N., Marwaha G. (2020). A 15-year profile of U.S. radiation oncology residency growth by geographic region, metropolitan size, and program size. Pract Radiat Oncol.

[6] Chowdhary M., Switchenko J.M., Sen N. (2019). The impact of graduates' job preferences on the current radiation oncology job market. Int J Radiat Oncol Biol Phys.

[7] Chowdhary M., Chhabra A.M., Switchenko J.M. (2017). Domestic job shortage or job maldistribution? A geographic analysis of the current radiation oncology job market. Int J Radiat Oncol Biol Phys.

[8] Association of American Medical Colleges Electronic residency application service statistics. https://www.aamc.org/eras-statistics-2019.

[9] Desantis C.E., Miller K.D., Sauer A.G. (2019). Cancer statistics for African Americans, 2019. CA Cancer J Clin.

[10] Siegel R.L., Miller K.D., Jemal A. (2020). Cancer statistics, 2020. CA Cancer J Clin.

[11] Siminoff L.A., Graham G.C., Gordon N.H. (2006). Cancer communication patterns and the influence of patient characteristics: Disparities in information-giving and affective behaviors. Patient Educ Couns.

[12] Boyd R.W. (2019). The case for desegregation. Lancet.

[13] Chapman C.H., Gabeau D., Pinnix C.C., Deville C., Gibbs I.C., Winkfield K.M. (2020). Why racial justice matters in radiation oncology. Adv Radiat Oncol.

[14] Hammoud M.M., Standiford T., Carmody J.B. (2020). Potential implications of COVID-19 for the 2020-2021 residency application cycle. JAMA.

[15] Jang S., Rosenberg S.A., Hullet C., Bradley K.A., Kimple R.J. (2018). Value of elective radiation oncology rotations: How many is too many?. Int J Radiat Oncol Biol Phys.

